# Reproductive Outcomes in Women Born With Low Birth Weight, Preterm or Small for Gestational Age: A Population‐Based Register Study

**DOI:** 10.1111/1471-0528.18350

**Published:** 2025-09-01

**Authors:** Susanne Liffner, Louise Möller, Marie Bladh, Elizabeth Nedstrand

**Affiliations:** ^1^ Department of Obstetrics and Gynaecology in Linköping and Division of Children's and Women's Health, Department of Biomedical and Clinical Sciences, Faculty of Medicine and Health Sciences Linköping University Linköping Sweden

**Keywords:** low birthweight, MAR treatment, preterm, reproductive pattern, small for gestational age

## Abstract

**Objective:**

To evaluate if the reduced likelihood of reproducing among women born small for gestational age (SGA), preterm (PT) or with low birth weight (LBW) persists towards the end of their reproductive era and to evaluate their use of medically assisted reproduction (MAR).

**Design:**

Population‐based register study.

**Setting:**

Sweden.

**Population/Sample:**

Women born in Sweden between 1973 and 1993 (*n* = 1 000 148).

**Methods:**

Data was retrieved from registers, among others the Swedish Medical Birth Register. The study population was stratified into two cohorts, born 1973 to 1983 and 1984 to 1993. The likelihood of giving birth was analysed using a Cox Proportional Hazards model.

**Main Outcome Measures:**

Giving birth to at least one child.

**Results:**

Women born SGA, PT or LBW had a lower probability of giving birth (presented as HR), adjusted for sociodemographic factors, with 95% CI: SGA: HR 0.94 (0.93–0.96); PT: HR 0.95 (0.94–0.96); and LBW: HR 0.90 (0.88–0.92) but no reduction was seen for women in the younger cohort born SGA (HR 0.98 [0.95–1.01]). Women born SGA or LBW had a lower chance of childbirth after MAR (SGA 80.3%, LBW 80.0%) compared to women born with normal weight (84.6%, *p* < 0.001) as well as an increased risk of being treated with oocyte donation (SGA 5.5% vs. 2.5%, *p* < 0.001; LBW 5.0% vs. 2.5%, *p* < 0.001).

**Conclusion:**

The decreased probability of parenthood for women born LBW, SGA, or preterm was even more evident after a longer follow‐up time. When pursuing MAR with their own oocytes, they had a lower chance of conceiving.

## 1 Introduction

Previous Swedish national register studies have shown that being born preterm (PT), with low birthweight (LBW) or small for gestational age (SGA), was associated with an altered reproductive pattern [1, 2, 3]. A reduced likelihood of childbearing was associated with women born with LBW or PT while women born SGA were more likely to give birth at a young age, but their probability of childbirth diminished over time [1, 2]. Women with a female infertility factor were more likely to be born LBW or SGA, suggesting an increased risk of infertility for women with non‐optimal birth characteristics [4], even though a Danish study found no association between women's birth weight for gestational age and infertility [5]. Studies of individuals born very PT (< 32 weeks' gestation) or with very LBW (< 1500 g) did not show an association with a lowered probability of becoming a parent in young adults (< 30 years) but seem to reduce fertility in older cohorts [6, 7].

Since the 90s there has been a theory stating that the function of the reproductive organs and hormone levels/production in adulthood are altered by events during foetal life [8, 9, 10, 11, 12, 13]. The SGA‐born women and men seem to suffer from several biological and psychological problems affecting, for example, the development of the nervous system and the reproductive organs or develop metabolic pathologies that might affect their future reproductive capacity [14, 15, 16, 17, 18, 19]. In the beginning of 2000s, Ibanez et al. found that the ovulation rate in adolescence was lower in females born SGA in comparison to their counterparts born with normal weight, but that they entered puberty and menarche at an earlier age [9, 11]. However, in a recent study from Sweden, no significant difference in reproductive hormones between women born with very low birthweight (VLBW) and women born at term was found, although the VLBW group of women reported menarche 1.5 years later than the control group [8]. Additionally, women with LBW enter menopause earlier than those with higher birthweight, and LBW has been associated with premature ovarian insufficiency, suggesting a reproductive period shorter than average [20, 21].

The primary aim of this study was to investigate the likelihood of giving birth in a group of women born with non‐optimal birth characteristics and to follow up on whether the previously reported reproductive pattern in women born SGA, preterm, or with LBW remained with a longer follow‐up time, now including a younger cohort. The second aim was to study the need for medically assisted reproduction (MAR) treatment, including the need for donated oocytes, in women born SGA, preterm and LBW.

## 2 Methods

### 2.1 Data Sources

The study is based on information from several Swedish national registers.

*The Swedish Medical Birth Register (MBR)* was established in 1973 and includes information regarding 99% of births in Sweden since then. The register contains information regarding prenatal care, delivery and neonatal care as well as maternal chronic diseases [22].

*The Cause of Death Register* was established in 1952. It includes information about the cause of death for Swedish residents, including those who pass away abroad. Infants dying during childbirth are not included. Since 2012 all deaths occurring in Sweden have been registered, including those of non‐Swedish residents. The coverage rate is close to 100% [23].

*The Total Population Register* includes information about a person's citizenship, country of birth, marital status, migration, death and birth. Some over‐coverage exists due to missing reports of death and immigration [20, 24].

*The Multi‐Generation Register* is based on the Total Population Register and includes all people born in Sweden since 1932 as well as those who have been registered in Sweden at some point since 1961. It is used to identify the parents of the index persons [25].

*The Education Register* was established in 1985 and includes information on the highest level of education and the year of completion. The register is limited to individuals between the age of 16 and 74 years. Before 1985, information about education was retrieved from the 1970 Population and Housing Census [26].

*The Swedish National Quality Register of Assisted Reproduction* (Q‐IVF) contains information on almost all MAR treatments in Sweden from 2007 to onward, including both public and private fertility clinics [27].

### 2.2 Study Population

**FIGURE 1:**
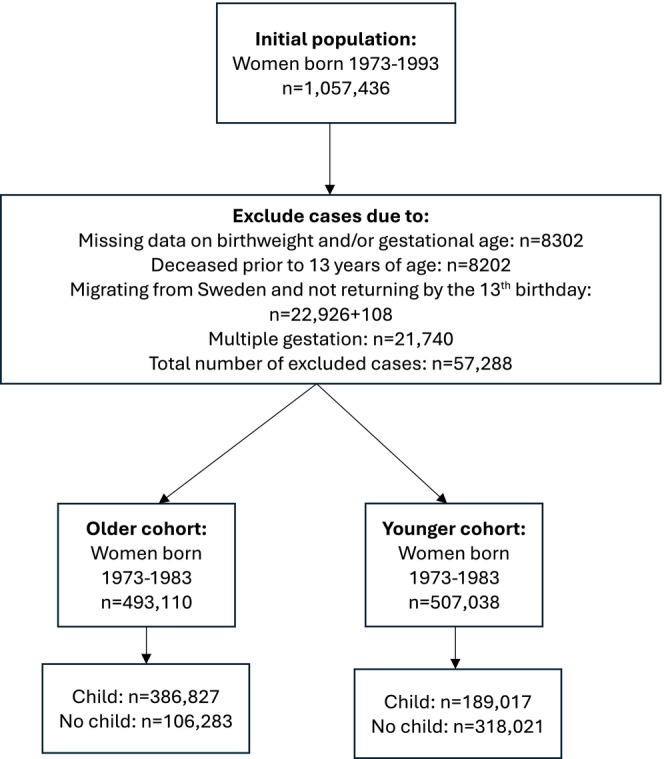
Flowchart describing population size, exclusion and inclusion conditions of the study population.

The study population was a cohort of 1 000 148 women born in Sweden between 1973 and 1993 who were alive and living in Sweden at the age of 13 years, that is, persons who had either died before 13 years of age or who had emigrated and not returned prior to their 13th birthday were excluded, as were women with missing values on either birthweight or gestational age. Only singleton births were included. The population was stratified into two groups, where the first group consisted of women born between 1973 and 1983 (older cohort) who were approaching the end of their reproductive period, while women born between 1984 and 1993 were assigned to the second group (the younger cohort). Both groups were followed from birth until 2018, with respect to having given birth to at least one child. A flowchart of the study population size and inclusion and exclusion criteria is displayed in Figure 1.

In the older cohort a sub‐group of women with any registered treatment at an infertility clinic in Sweden between 2007 and 2018 was used to investigate the frequency and type of MAR treatments in women with non‐optimal birth characteristics compared to optimal birth characteristics.

### 2.3 Variable Definition

**FIGURE 2:**
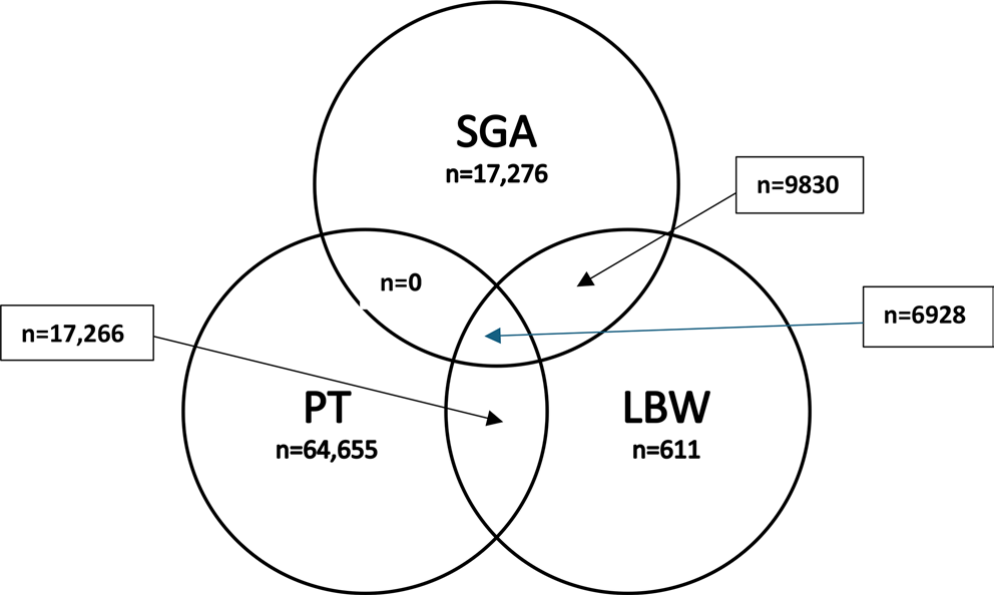
Display of the overlap of SGA, PT and LBW.

SGA was defined as gender and gestational age‐specific birthweight below 2 SD from the mean birthweight according to Swedish standards [28]. The WHO definition of SGA, a birth weight below the 10th percentile, was also used as a variable for comparative reasons [29]. LBW was defined as ≤ 2500 g while very low birth weight (VLBW) was defined as < 1500 g. Preterm birth (PT) was defined as being born before gestational Weeks 37 and very preterm birth (VPT) was defined as being born before gestational Weeks 32. There was a considerable overlap on LBW, PT and SGA as displayed in Figure 2.

The educational level of the index women as well as their parents was coded into elementary, high school, or college/university. Marital status was coded into married/cohabiting, single and divorced/widowed. Variables associated with MAR treatment include the type of first treatment categorised into IVF (in vitro fertilisation) or ICSI (intra‐cytoplasmic sperm injection), the use of donated oocytes or sperm cells, achieved pregnancy and live born infant, all coded into yes and no.

### 2.4 Statistical Analysis

Data on categorical variables is presented by numbers (*n*) and percent (%), whereas continuous variables are described by mean and SD. Initial analyses include Pearson's Chi‐square to analyse the association between having had a child and independent variables, while continuous outcomes are analysed using the Student's *t*‐test.

Further analyses regarding the likelihood of giving birth were performed using Cox's proportional hazards model, where hazard ratios (HR) and their 95% confidence intervals (CI) are presented. Sociodemographic background characteristics, that is, maternal and paternal educational level, mother's marital status, parents' country of origin, mother's parity and maternal age at childbirth were treated as potential confounders and adjusted for, and results are presented both as crude and adjusted HR. Analyses were performed separately for each of the exposure variables (SGA 2 SD, SGA 10th percentile, PT, VPT, LBW and VLBW). Women exited the model either when they gave birth, emigrated, or died—whichever came first.

Descriptive analyses pertaining to MAR treatment outcomes such as achieving a pregnancy or giving birth to a live born child were restricted to women in the older cohort (women born 1973–1983) to ensure that the maximum number of women had the opportunity, if required, to achieve pregnancy, to use MAR.

All analyses were performed using IBM SPSS, version 28 (IBM Inc., Armonk, NY, USA). Statistical significance was defined as *p* < 0.05 (two‐sided).

### 2.5 Ethical Consideration

All data was retrieved from population‐based registers supplied by Statistics Sweden and the National Board of Health and Welfare.

The study has been approved by the Ethical Review Board of Linköping, Sweden (2017/513‐31). All data has been pseudonymized and informed consent from each individual was not necessary to obtain.

No patient or public involvement took place in the study. Core outcome sets for neonatal care do not include reproductive health/fertility as a core outcome and could not be used.

The study followed the STROBE reporting guideline [30].

## 3 Results

The women in the study population were on average 27.6 years old when having their first child, 28.6 years old in the older cohort and 25.5 years old in the younger cohort. The lower mean age at first birth reflects that most of the women in the younger cohort had not started having children yet. Overall, 58% had at least one child, where the proportion was about twice as high in the older cohort (78%) compared to the younger cohort (37%), Table S1. Sociodemographic data and infertility treatments in relation to being born SGA, PT or with LBW are presented for the two age cohorts in Tables 1 and 2 respectively. In Table S1 the same data are presented in relation to whether the women have had a child or not. In Sweden, cohabiting without being married is very common and the registration in national registers has changed over the years. The maternal marital status therefore seems to differ greatly between the age cohorts as non‐married women were registered as single when giving birth up until 1987.

**TABLE 1:** Women born 1973 to 1983, non‐optimal birth characteristics in relation to sociodemographic and medical background factors.

	SGA 2 SD			SGA 10th percentile			PT			LBW		
	No	Yes	*p*	No	Yes	*p*	No	Yes	*p*	No	Yes	*p*
	*n* (%)	*n* (%)		*n* (%)	*n* (%)		*n* (%)	*n* (%)		*n* (%)	*n* (%)	
*Parents*												
Maternal educational level			< 0.001			< 0.001			< 0.001			< 0.001
Elementary	81 852 (19.0)	4020 (23.4)		75 892 (18.8)	9980 (22.1)		78 404 (19.0)	7468 (21.5)		82 541 (19.0)	3331 (22.9)	
High school	209 581 (48.6)	8835 (51.4)		195 477 (48.5)	22 939 (50.8)		201 212 (48.6)	17 204 (49.5)		210 979 (48.6)	7437 (51.1)	
College/University	139 823 (32.4)	4339 (25.2)		131 915 (32.7)	12 247 (27.1)		134 086 (32.4)	10 076 (29.0)		140 379 (32.4)	3783 (26.0)	
Paternal educational level			< 0.001			< 0.001			< 0.001			< 0.001
Elementary	107 007 (27.6)	4821 (31.2)		99 543 (27.5)	12 285 (30.3)		102 683 (27.7)	9145 (29.3)		107 851 (27.7)	3977 (30.5)	
High school	171 816 (44.4)	7185 (46.6)		160 349 (44.3)	18 652 (36.0)		164 971 (44.4)	14 030 (45.0)		173 017 (44.4)	5984 (45.9)	
College/University	108 218 (28.0)	3429 (22.2)		102 031 (28.2)	9616 (23.7)		103 621 (27.9)	8026 (25.7)		108 568 (27.9)	3079 (23.6)	
Mother born in a non‐Nordic country	26 187 (5.5)	1229 (6.1)	< 0.001	24 397 (5.5)	3019 (5.9)	0.001	24 996 (5.5)	2420 (6.1)	< 0.001	26 377 (5.5)	1039 (6.1)	0.001
Father born in a non‐Nordic country	22 633 (4.8)	1153 (5.8)	< 0.001	21.075 (4.8)	2711 (5.3)	< 0.001	21 635 (4.8)	2151 (5.5)	< 0.001	22 770 (4.8)	1016 (6.0)	< 0.001
Marital status			< 0.001			< 0.001			< 0.001			< 0.001
Married	289 227 (68.9)	10 733 (60.3)		271 656 (69.3)	28 304 (63.0)		276 712 (68.7)	23 248 (67.1)		290 761 (68.7)	9289 (62.8)	
Single	122 969 (29.3)	6640 (37.3)		113 276 (28.9)	15 333 (35.8)		119 044 (29.5)	10 565 (30.5)		124 483 (29.4)	5126 (34.6)	
Divorced/Widow	7632 (1.8)	431 (2.4)		7072 (1.8)	991 (2.2)		7253 (1.8)	810 (2.3)		7683 (1.8)	380 (2.6)	
Primiparous	198 481 (42.0)	10 819 (54.0)	< 0.001	182 171 (41.2)	27.129 (52.8)	< 0.001	191 557 (42.2)	17 743 (44.9)	< 0.001	200 085 (42.2)	8495 (49.9)	< 0.001
Maternal age at childbirth			< 0.001			< 0.001			< 0.001			< 0.001
13–19	26 559 (5.6)	1598 (8.0)		24 273 (5.5)	3884 (7.6)		252 575 (5.6)	2882 (7.3)		26 814 (5.6)	1343 (7.9)	
20–26	208 162 (44.2)	9611 (48.0)		193 731 (43.9)	25 042 (48.7)		202 543 (44.7)	16 230 (41.1)		211 447 (44.4)	7326 (43.1)	
27–33	189 545 (40.1)	6951 (34.7)		178 286 (40.4)	18 210 (35.4)		181 394 (40.0)	15 102 (38.2)		190 266 (40.0)	6230 (36.6)	
> 33	47 809 (10.1)	1875 (9.4)		45 416 (10.3)	4268 (8.3)		44 394 (9.8)	5290 (13.4)		47 574 (10.0)	2110 (12.4)	
*Index women*												
Level of education			< 0.001			< 0.001			< 0.001			< 0.001
Elementary	22 212 (5.0)	1411 (7.5)		20 352 (4.9)	3271 (6.8)		21 274 (5.0)	2349 (6.4)		22 431 (5.0)	1192 (7.5)	
High school	157 775 (35.3)	7887 (42.1)		146 234 (35.1)	19 428 (40.2)		151 914 (35.5)	13 748 (37.2)		159 201 (35.4)	6461 (40.9)	
College/University	266 465 (59.7)	9424 (50.3)		250 312 (60.0)	25 577 (53.0)		255 000 (59.6)	20 889 (56.5)		267 753 (59.6)	8136 (51.5)	
Ever married or cohabiting, yes	252 853 (53.4)	10 481 (52.4)	0.002	236 140 (53.5)	27 194 (52.9)	**0.016**	243 142 (53.6)	20 192 (51.1)	**< 0.001**	254 785 (53.5)	8549 (50.3)	**< 0.001**
Child, yes	371 593 (78.5)	15 234 (76.0)	< 0.001	346 929 (78.5)	39 898 (77.6)	< 0.001	357 171 (78.7)	29 656 (75.1)	< 0.001	374 361 (78.6)	12 466 (73.3)	< 0.001
*Infertility treatment*												
Q‐IVF, yes	19 339 (4.1)	785 (3.9)	0.243	18 014 (4.1)	2110 (4.1)	0.774	18 624 (4.1)	1500 (3.8)	0.003	19 509 (4.1)	615 (3.6)	0.002
Donated oocyte^a^, yes	484 (2.5)	43 (5.5)	< 0.001	439 (2.4)	88 (4.2)	< 0.001	476 (2.6)	51 (3.4)	0.049	496 (2.5)	31 (5.0)	< 0.001

*Note:* SGA 2 SD = small for gestational age (birth weight < −2 SD of mean weight for gestational age); SGA 10th percentile = small for gestational age (birthweight below the 10th percentile of mean weight for gestational age); PT = preterm (birth before 37th gestational week); LBW = low birth weight (< 2500 g); Marital status: Married = ever registered as married in the Total Population Register (TPR); Single = never registered as married or cohabiting in the TPR; Q‐IVF = number and percentage of women registered in Q‐IVF, the Swedish National Quality Register of Assisted Reproduction. *p*‐values < 0.05 are considered to indicate statistical significance.

^a^ Limited to women who have undergone infertility treatment.

**TABLE 2:** Women born 1984–1993, non‐optimal birth characteristics in relation to sociodemographic and medical background factors.

	SGA			SGA 10th percentile			PT			LBW		
	No	Yes	*p*	No	Yes	*p*	No	Yes	*p*	85 514 (No)	Yes	*p*
	*n* (%)	*n* (%)		*n* (%)	*n* (%)		*n* (%)	*n* (%)		*n* (%)	*n* (%)	
*Parents*												
Maternal educational level			< 0.001			< 0.001			< 0.001			< 0.001
Elementary	52 860 (11.1)	1953 (14.8)		39 438 (11.0)	5375 (14.4)		48 595 (11.0)	6218 (13.3)		52 357 (11.1)	2456 (14.8)	
High school	241 349 (50.8)	7035 (53.4)		228 583 (50.7)	19.810 (53.0)		223 703 (50.6)	24 681 (52.6)		239 555 (50.7)	8829 (53.4)	
College/University	181 265 (38.1)	4175 (31.7)		173 251 (38.4)	12 189 (32.6)		169 433 (38.4)	16 007 (34.1)		180 180 (38.2)	5260 (31.8)	
Paternal educational level			< 0.001			< 0.001			< 0.001			< 0.001
Elementary	86 014 (19.0)	2725 (21.9)		81 173 (18.9)	7506 (21.4)		79 540 (18.9)	9199 (20.6)		85 349 (19.0)	3390 (21.6)	
High school	231 906 (51.3)	6492 (52.2)		219 822 (51.2)	18.576 (52.7)		215 158 (51.2)	23 240 (52.2)		230 203 (51.3)	8195 (52.3)	
College/University	134 074 (29.7)	3225 (25.9)		128 160 (29.9)	9139 (25.9)		125 190 (29.8)	12 109 (27.2)		133 222 (29.7)	4077 (26.0)	
Mother born in a non‐Nordic country	23 446 (4.8)	765 (5.5)	< 0.001	22 084 (4.7)	2127 (5.4)	< 0.001	2177 (4.8)	2434 (4.9)	0.085	23 285 (4.8)	926 (5.3)	0.002
Father born in a non‐Nordic country	22 090 (4.5)	766 (5.5)	< 0.001	20 799 (4.5)	2057 (5.3)	< 0.001	20 506 (4.5)	2350 (4.8)	0.003	21 886 (4.5)	970 (5.5)	< 0.001
Maternal marital status at time of childbirth			< 0.001			< 0.001			< 0.001			< 0.001
Married	436 040 (94.6)	11 794 (91.2)		414 174 (94.7)	33 660 (92.0)		405 436 (94.7)	42 398 (93.3)		433 176 (94.6)	14 658 (91.7)	
Single	8900 (1.9)	452 (3.5)		8213 (1.9)	1139 (3.1)		8243 (1.9)	1109 (2.4)		8853 (1.9)	499 (3.1)	
Divorced/Widow	15 890 (3.4)	689 (5.3)		14 807 (3.4)	1772 (4.8)		14 666 (3.4)	1913 (4.2)		157,52 (3.4)	827 (5.2)	
Primiparous	201 078 (40.8)	8027 (57.3)	< 0.001	187 671 (40.1)	21 434 (54.5)	< 0.001	186 588 (40.8)	22 517 (45.6)	< 0.001	199 781 (40.8)	9324 (52.4)	< 0.001
Maternal age at childbirth			< 0.001			< 0.001			< 0.001			< 0.001
13–19	14 091 (2.9)	518 (3.7)		13 110 (2.8)	1499 (3.8)		12 827 (2.8)	1782 (3.6)		13 939 (2.8)	670 (3.8)	
20–26	187 718 (38.1)	5420 (38.7)		177 231 (37.9)	15 907 (40.4)		174 400 (38.1)	18 738 (38.0)		186 553 (38.1)	6585 (37.4)	
27–33	216 490 (43.9)	5595 (40.0)		206 005 (44.0)	16 080 (40.9)		102 950 (44.1)	20 135 (40.8)		215 107 (44.0)	6978 (39.6)	
34—	74 740 (15.2)	2466 (17.6)		71 348 (15.3)	5858 (14.9)		68 516 (15.0)	8690 (17.6)		73 813 (15.1)	3393 (19.2)	
*Index women*												
Level of education			< 0.001			< 0.001			< 0.001			< 0.001
Elementary	28 869 (6.1)	1174 (8.9)		26 992 (6.0)	3051 (8.2)		26 599 (6.0)	3444 (7.3)		28 593 (6.1)	1450 (8.7)	
High school	198 830 (41.9)	5857 (44.5)		188 065 (41.8)	16 622 (44.5)		184 722 (42.0)	19 965 (42.4)		197 338 (41.9)	7349 (44.2)	
College/University	246 394 (52.0)	6126 (46.6)		234 828 (52.2)	17 692 (47.3)		228 866 (52.0)	23 654 (50.3)		244 705 (52.0)	7815 (47.0)	
Ever married or cohabiting, yes	81 560 (16.5)	2397 (17.1)	0.069	77 234 (16.5)	6723 (17.1)	0.003	75 608 (16.5)	8349 (16.9)	0.023	81 141 (16.6)	2816 (16.0)	0.034
Child, yes	183 737 (37.3)	5280 (37.7)	0.277	173 870 (37.2)	15 147 (38.5)	< 0.001	170 496 (37.3)	18 521 (37.5)	0.219	182 658 (37.3)	6359 (36.1)	< 0.001
*Infertility treatment*												
Q‐IVF, yes	6144 (1.2)	190 (1.4)	0.243	5800 (1.2)	534 (1.4)	0.045	5706 (1.2)	628 (1.3)	0.621	6136 (1.3)	198 (1.1)	0.126
Donated oocyte^a^, yes	91 (1.5)	8 (4.2)	0.003	83 (1.4)	16 (3.0)	0.005	86 (1.5)	13 (2.1)	0.280	90 (1.5)	9 (4.5)	< 0.001

*Note:* SGA 2 SD = small for gestational age (birth weight < −2 SD of mean weight for gestational age); SGA 10th percentile = small for gestational age (birthweight below the 10th percentile of mean weight for gestational age); PT = preterm (birth before 37th gestational week); LBW = low birth weight (< 2500 g); Marital status: Married = ever registered as married in the Total Population Register (TPR); Single = never registered as married or cohabiting in the TPR; Q‐IVF = number and percentage of women registered in Q‐IVF, the Swedish National Quality Register of Assisted Reproduction. *p* < 0.05 are considered to indicate statistical significance.

^a^ Limited to women who have undergone infertility treatment.

In the older cohort, being born SGA, PT or with LBW were all associated with a lower likelihood of giving birth (SGA −2 SD: HR = 0.97, 95% CI = 0.95–0.99; Preterm: HR = 0.93, 95% CI = 0.92–0.94; low birthweight: HR = 0.90, 95% CI = 0.89–0.92), Table 3. When using the birth weight below the 10th percentile as the SGA variable, no difference in reproductive rate was detected, Table 3. These are crude HR estimates. To determine whether the reproductive pattern changed with a longer follow‐up, the estimates in the older cohort were compared to the numbers presented in a study by deKeyser et al. comprising the same women but then with a follow‐up until the end of 2006 [2]. When adjusted for the same socio‐economic background variables except for the year of birth, the HR estimates were lower for women born SGA and with LBW but not changed for women born PT, Table 3. In 2006, being born SGA did not seem to lower the reproductive rate (aHR 1.01, 95% CI 0.99–1.03) but with the longer time of follow‐up the aHR had decreased to 0.93 (95% CI 0.91–0.95). The aHR for women born LBW was now also lower, aHR 0.88 (95% CI 0.87–0.91) compared with 0.95 (95% CI 0.93–0.97) in 2006, Table 3. Additional adjusting for index women's relationship status and educational level did not alter the HR significantly, and these results are not presented.

**TABLE 3:** Hazard ratios of reproducing and corresponding 95% confidence intervals.

Cohort	SGA, 2 SD		SGA 10th percentile		PT		VPT		LBW		VLBW	
	HR (95% CI)	*p*	HR (95% CI)	*p*	HR (95% CI)	*p*	HR (95% CI)	*p*	HR (95% CI)	*p*	HR (95% CI)	*p*
Total population	0.98 (0.97–0.99)^a^	0.007	1.01 (1.00–1.02)^a^	0.002	0.95 (0.94–0.96)^a^	< 0.001	0.76 (0.72–0.79)^a^	< 0.001	0.92 (0.91–0.93)^a^	< 0.001	0.77 (0.74–0.81)^a^	< 0.001
	0.94 (0.93–0.96)^b^	< 0.001	0.98 (0.97–0.99)^b^	< 0.001	0.95 (0.94–0.96)^b^	< 0.001	0.75 (0.72–0.79)^b^	< 0.001	0.90 (0.88–0.92)^b^	< 0.001	0.78 (0.74–0.83)^b^	< 0.001
1984–1993	1.00 (0.97–1.02)^a^	0.863	1.03 (1.01–1.05)^a^	< 0.001	0.99 (0.97–1.00)^a^	0.138	0.78 (0.72–0.84)^a^	< 0.001	0.95 (0.93–0.97)^a^	< 0.001	0.80 (0.74–0.86)^a^	< 0.001
	0.98 (0.95–1.01)^b^	0.240	1.00 (0.98–1.02)^b^	0.940	0.97 (0.96–0.99)^b^	< 0.001	0.75 (0.69–0.81)^b^	< 0.001	0.93 (0.90–0.95)^b^	< 0.001	0.78 (0.72–0.85)^b^	< 0.001
1973–1983	0.97 (0.95–0.99)^a^	< 0.001	1.00 (0.99–1.01)^a^	0.643	0.93 (0.92–0.94)^a^	< 0.001	0.75 (0.71–0.79)^a^	< 0.001	0.90 (0.89–0.92)^a^	< 0.001	0.76 (0.81–0.81)^a^	< 0.001
	0.93 (0.91–0.95)^b^	< 0.001	0.98 (0.96–0.99)^b^	< 0.001	0.94 (0.92–0.95)^b^	< 0.001	0.75 (0.70–0.81)^b^	< 0.001	0.88 (0.87–0.91)^b^	< 0.001	0.78 (0.72–0.84)^b^	< 0.001
1973–1983^c^	1.01 (0.99–1.03)	0.437			0.94 (0.92–0.96)	< 0.001	0.81 (0.74–0.88)	< 0.001	0.95 (0.93–0.97)	< 0.001	0.80 (0.72–0.89)	< 0.001

*Note:* SGA 2 SD = small for gestational age (birth weight < −2 SD of mean weight for gestational age); SGA 10th percentile = small for gestational age (birthweight below the 10th percentile of mean weight for gestational age); PT = preterm (birth before 37th gestational week); VPT = very preterm (birth before 32nd gestational week); LBW = low birth weight (< 2500 g); VLBW = very low birth weight (< 1500 g). *p* < 0.05 are considered to indicate statistical significance.

Abbreviations: CI = confidence interval, HR = hazard ratio.

^a^ Crude estimates.

^b^ Estimates adjusted for maternal and paternal educational level, mother's marital status, parents' country of origin, mother's parity and maternal age at childbirth.

^c^ Estimates presented in a previously published paper by de Keyser et al. 2012 (adjusted for maternal and paternal educational level, mother's marital status, parents' country of origin, mother's parity and maternal age at childbirth and year of birth).

In the younger cohort, LBW was shown to decrease the likelihood of giving birth (aHR 0.93, 95% CI 0.90–0.95) as well as PT birth to a lesser extent (aHR 0.97, 95% CI 0.96–0.99), Table 3. Being born SGA did still not lower the reproductive rate in younger ages.

Women born with VPT had a similar adjusted reproductive rate in both age cohorts, with HR of 0.75 (older cohort 95% CI 0.70–0.81; younger cohort 95% CI 0.69–0.81); the aHR in 2006 was 0.81 (95% CI 0.74–0.88). The reproductive rate for women born VLBW was also the same regardless of age cohort (aHR 0.78, older cohort 95% CI 0.72–0.84, younger cohort 95% CI 0.72–0.85) and tended to be lower than in 2006, although not statistically significant (aHR 0.80, 95% CI 0.72–0.89), Table 3.

Treatments with MAR were not generally more common in women born SGA, PT, or with LBW, but women born SGA or LBW using MAR had a larger need for donated oocytes compared with controls (women 1973–1983 SGA 5.5% vs. 2.5% and LBW 5.0% vs. 2.5%; both *p* < 0.001; women 1984–1993 SGA 4.2% vs. 1.5% (*p* = 0.003), and LBW 4.5% vs. 1.5% (*p* < 0.001)), Tables 1 and 2. Women born VPT or VLBW were less often patients at IVF clinics and the absolute number of women treated with MAR was small (50 women born VPT and 44 women born VLBW), Table S2. The proportion of VLBW women in need of donated oocytes was larger (9.1% (4/44 individuals) compared with 2.6% of women not VLBW, *p* = 0.007), Table S2. No difference in the use of donated sperm was found, nor in the proportion of ICSI cycles (data not shown).

To further investigate the use of MAR treatments and its relation to non‐optimal birth characteristics, sub‐group analyses on women in the older cohort who had gone through IVF were performed. Beyond the increased need for donated oocytes reported in Table 1 and 2 for women born SGA or with LBW, they were also less likely to have given birth to a child, Table 4. Of the women born SGA, 80.3% gave birth after MAR compared with 84.6% of the women born with normal birth weight at term (*p* < 0.001) and the proportions were similar for women born with LBW (80.0% compared with 84.6% of women born with normal BW, *p* = 0.002). Sub‐analyses of childbirth in relation to origin of oocytes showed that 80.7% of the women born SGA gave birth after MAR using own oocytes (compared with 85.0% of women born non‐SGA) but when donated oocytes were used, the chance of childbirth was higher for women born SGA (72.1% vs. 70.9%, *p* < 0.001).

**TABLE 4:** Assisted reproduction treatments and outcome in women born between 1973 and 1983, limited to women who had undergone infertility treatment.

Variable	SGA, 2 SD			SGA 10th percentile			Preterm			Very preterm			Low birth weight			Very Low birth weight		
	AGA	SGA	*p*	AGA	SGA	*p*	Term	PT	*p*	No	Yes	*p*	Normal weight	LBW	*p*	No	Yes	*p*
	*n* (%)	*n* (%)		*n* (%)	*n* (%)		*n* (%)	*n* (%)		*n* (%)	*n* (%)		*n* (%)	*n* (%)		*n* (%)	*n* (%)	
Donated oocyte			< 0.001			< 0.001			0.049			0.378			< 0.001			0.028
No	18 855 (97.5)	742 (94.5)		17 575 (97.6)	2022 (95.8)		18 148 (97.4)	1449 (96.6)		19 549 (97.4)	48 (96.0)		19 013 (97.5)	584 (95.0)		19 557 (97.4)	40 (90.9)	
Yes	484 (2.5)	43 (5.5)		439 (2.4)	88 (4.2)		476 (2.6)	51 (3.4)		525 (2.6)	2 (4.0)		496 (2.5)	31 (5.0)		523 (2.6)	4 (9.1)	
Child			< 0.001			0.050			0.386			0.385			0.002			0.189
No	2975 (15.4)	155 (19.7)		2771 (15.4)	359 (17.0)		2885 (15.5)	245 (16.3)		3120 (15.5)	10 (20.0)		3007 (15.4)	123 (20.0)		3120 (15.5)	10 (22.7)	
Yes	16 364 (84.6)	630 (80.3)		15 243 (84.6)	1751 (83.0)		15 739 (84.5)	1255 (83.7)		16 954 (84.5)	40 (80.0)		16 502 (84.6)	492 (80.0)		16 960 (84.5)	34 (77.3)	

*Note:* SGA 2 SD = small for gestational age (birth weight < −2 SD of mean weight for gestational age); SGA 10th percentile = small for gestational age (birthweight below the 10th percentile of mean weight for gestational age); PT = preterm (birth before 37th gestational week); LBW = low birth weight (< 2500 g). AGA = average for gestational age (birth weight within 2 SD of the mean weight for gestational age); Child = number (percentage) of women in whom assisted reproduction treatment led to the birth of a child; Donated oocyte = number (percentage) of women undergoing treatment with donated sperm cells; Q‐IVF = Swedish National Quality Register of Assisted Reproduction.  *p*‐values < 0.05 are considered to indicate statistical significance.

## 4 Discussion

The results from this national cohort study show that women born preterm, SGA, or with LBW had a lower probability of giving birth compared to women in the same age groups born at term, but for women born SGA, this is not shown in the younger cohort, supporting the previous suggestion of a different pattern of reproduction for women born SGA. The results are in line with earlier studies from 2000 [1, 2] on a subset of the cohort used in the current study, but now with a longer follow‐up period.

The estimates of the HR were consistently lower for women born LBW even though not always reaching statistical significance. Comparing the reproductive rates when the older and the younger cohort were at the same age using data from deKeyser et al. for the cohort born 1973–1983 (aHR 0.95, 95% CI 0.93–0.97), the impaired fertility in women born LBW was now more pronounced in the younger cohort (aHR 0.93, 95% CI 0.90–0.95) [2]. For women born PT, a tendency towards a higher reproductive rate was present for the younger cohort (aHR 0.97, 95% CI 0.96–0.99) compared to the older cohort at the same age (aHR 0.94, 95% CI 0.92–0.96) [2]. This could suggest that LBW is more detrimental for reproduction than prematurity, maybe explained by improved neonatal care of children born PT over the years rendering the adults born PT more able to reproduce even if the negative effects on reproduction exerted by LBW are still present. On the other hand, the smaller groups of women born VPT and VLBW had low reproductive rates that did not differ in relation to age cohort, suggesting a greater impact on reproduction with a more severe prematurity or lower birth weight.

Women born SGA, PT or LBW were patients at an IVF clinic to a similar or lower extent than women born with normal birth weight even though they more often were childless (Tables 1 and 2). Besides the possible explanation that women born with non‐optimal birth characteristics more often were childless by choice, it could also reflect an inability to go through MAR treatments among the women born LBW, SGA or PT. They may have other health issues, preventing them from trying to pursue a pregnancy, and they had also more rarely been married or cohabiting (Tables 1 and 2). Among the women born SGA or LBW that were patients at an IVF clinic, the use of oocyte donation was more common (Tables 1 and 2), indicating oocyte dysfunction or a lower ovarian reserve. Similarly, a population‐based study found that men born SGA were more often diagnosed with infertility and needed ICSI or sperm donation more often than men born AGA, suggesting an increased risk of sperm dysfunction for these men [3]. The reason for lower reproductive capacity is, of course, multidimensional but some evidence, namely findings of hormonal and/or organ deficiency among women born SGA, preterm or with LBW, might be an explanatory factor [9, 11]. It has previously been shown that women with non‐optimal birth characteristics are more prone to premature ovarian insufficiency, hormonal abnormalities and early menopause [20, 21] and this could explain the increased need for oocyte donation.

Unfortunately, information on the underlying reason why women needed donated oocytes to achieve pregnancy was unavailable in the current study. This removed the possibility to control for causes such as Turner's syndrome and other chromosomal abnormalities. Chromosomal abnormalities are also associated with SGA, PT and LBW and could be a confounding factor. In general, MAR treatments are quite generously covered within the Swedish health care system, including up to three publicly financed cycles of IVF or oocyte donation if needed due to medical conditions. At the same time must the risk of complications with a pregnancy be reasonable in order to be accepted for MAR treatments, particularly treatments with donated gametes. Women with Turner's syndrome for example, can only be accepted for oocyte donation if their cardiac condition is good [31].

Reasons for a lower reproduction rate might also be explained by the increase of neurological and psychological disorders found among women born prematurely. This could lead to problems that could potentially make it harder to find a partner and be in a relationship [32, 33, 34] and is probably one of the explanations for the increased likelihood of being single in the current study.

The study by Ekholm et al. showed that women born SGA had a significantly higher reproduction rate by the time they reached 25–27 years of age compared to those born AGA. The study also showed a trend towards decreasing reproductive rate with age for those with non‐optimal birth characteristics [1]. Other studies have found similar patterns in women born PT or LBW [6, 7]. This tendency could be explained by life history theory, where a higher mortality risk (e.g., PT, LBW or SGA) may induce a faster strategy of reproduction (i.e., conceiving earlier in life) [7, 35]. In the current study, it was found that the population of women born SGA indeed had a lower rate of reproduction after a longer follow‐up time even if no difference was found in the younger cohort, supporting the theory of a different reproductive pattern compared to women born at term.

The definition of SGA used in this study, that is, a gender and gestational age‐specific birth weight below 2 SD from the mean birthweight according to Swedish standards, is often used in the Nordic countries and was also recommended in a consensus statement [36, 37]. This can be considered a strength since it is a stricter definition of SGA than the 10th percentile definition according to WHO [29]. The use of different definitions makes it difficult to compare results from different research studies, and in the present study, data using the 10th percentile definition were also reported for comparative reasons.

The strengths of the present study include the use of data from national validated registers. This limits the risk of recall bias. Also, since it is a population‐based cohort study, the demographics in the current study follow those of the general Swedish female population. Using two age cohorts made it possible for a longer follow‐up time for the older age group, that is, until they were leaving the reproductive period in life, whereas women in the younger cohort were just entering their ‘peak’ reproductive era. Comparisons of reproductive rate at the same age but in relation to birth year were also possible with this study design.

The limitation of this study was the unavailability of information on potential confounders such as smoking, body mass index, alcohol, work situation, miscarriages, legal abortions, as well as partner status. Alcohol consumption and smoking habits are not consistently reported in the Medical Birth Register and the other factors are not covered in the register. See Figure S1 for a directed acyclic graph on potential confounding and mediation. The lack of MAR data prior to 2007, as the register was not initiated until 2007, is also a limitation.

## 5 Conclusion

Women born with LBW or PT were shown to have a lower probability of giving birth compared to women born with normal birth weight at term. The reduction of the reproduction rate was more evident after a longer follow‐up time. Women born SGA showed a lower probability of giving birth over time, although they initially had a normal reproduction rate at a younger age.

The proportion of women born SGA/LBW/PT treated at IVF clinics was similar to or lower than that of women born AGA in general, but they had an increased likelihood of being treated with oocyte donation. When pursuing IVF or ICSI with their own oocytes, women born SGA or LBW had a lower chance of having a child, whereas their chance of having a baby after oocyte donation was at least as good as the chance for women born AGA.

## Author Contributions

S.L., M.B., L.M. and E.N. conceptualised, planned and designed the study. M.B. was responsible for analyses of the data. S.L. and M.B. were primarily responsible for writing the paper. All authors were involved in interpreting the results, revising the paper with respect to content and approved the final version of the manuscript for submission.

## Ethics Statement

The study has been approved by the Ethical Review Board of Linköping, Sweden (2017/513‐31), 22 November 2017.

## Conflicts of Interest

The authors declare no conflicts of interest.

## Supporting information


**Table S1:** Descriptive data of women born between 1973 and 1983 and between 1984 and 1993 in Sweden.
**Table S2:** Very preterm birth and very low birth weight in relation to sociodemographic and medical background factors for women born between 1973 and 1983 and between 1984 and 1993 in Sweden.
**Figure S1:** Directed acyclic graph on potential confounding and mediation.

## Data Availability

The data that support the findings of this study may be available on request from the corresponding author. The data are not publicly available due to privacy or ethical restrictions.
