# Supplementary material for: Reproductive Outcomes in Women Born With Low Birth Weight, Preterm or Small for Gestational Age: A Population‐Based Register Study
Source: BJOG. 2025 Sep 1;133(1):165–77. doi: 10.1111/1471-0528.18350 (PMC12676200; doi:10.1111/1471-0528.18350)

Table S1. Descriptive data of women born between 1973 and 1983 and between 1984 and 1993 in Sweden.

|  | **Women born between 1973 and 1983**  **(n=493,110)** | | | **Women born between 1984 and 1993**  **(n=507,038)** | | |
| --- | --- | --- | --- | --- | --- | --- |
|  | **No child**  **(n=106,283)** | **Child**  **(n=386,827)** |  | **No child**  **(n=318,021)** | **Child**  **(n=189,017)** |  |
|  | **n (%)** | **n (%)** | **p-value** | **n (%)** | **n (%)** | **p-value** |
| **Parents** |  |  |  |  |  |  |
| Maternal educational level |  |  | <0.001 |  |  | <0.001 |
| Elementary | 17,045 (18.1) | 68,827 (19.4) |  | 28,525 (9.3) | 26,288 (14.4) |  |
| High school | 43,940 (46.6) | 174,476 (49.3) |  | 146,719 (47.9) | 101,665 (55.8) |  |
| College/University | 33,250 (35.3) | 110,912 (31.3) |  | 131,210 (42.8) | 54,230 (29.8) |  |
| Paternal educational level |  |  | <0.001 |  |  | <0.001 |
| Elementary | 21,426 (25.5) | 90,402 (28.4) |  | 47,792 (16.3) | 40,947 (23.8) |  |
| High school | 36,536 (43.5) | 142,465 (44.7) |  | 144,366 (49.4) | 94,032 (54.7) |  |
| College/University | 26,028 (31.0) | 85,619 (26.9) |  | 100,317 (34.3) | 36,982 (21.5) |  |
| Maternal region of birth |  |  | <0.001 |  |  | <0.001 |
| Non-Nordic country | 7,471 (7.0) | 19,945 (5.2) |  | 15,594 (4.9) | 8,617 (4.6) |  |
| Nordic country | 98,788 (93.0) | 366,878 (94.8) |  | 312,413 (95.1) | 180,399 (95.4) |  |
| Paternal region of birth |  |  | <0.001 |  |  | .404 |
| Non-Nordic country | 6,317 (6.0) | 17,469 (4.5) |  | 14,273 (4.5) | 8,583 (4.6) |  |
| Nordic country | 99,141 (94.0) | 367,442 (95.5) |  | 302,256 (95.5) | 179,653 (95.4) |  |
| Marital status (mother) |  |  | <0.001 |  |  | <0.001 |
| Married | 64,541 (70.9) | 234,509 (67.9) |  | 279,295 (94.9) | 168,539 (84.0) |  |
| Single | 25,020 (27.1) | 104,589 (30.3) |  | 4739 (1.6) | 10,401 (3.5) |  |
| Divorced/Widow | 1833 (2.0) | 6230 (1.8) |  | 10,401 (3.5) | 6178 (3.4) |  |
| Parity |  |  | 0.045 |  |  | <0.001 |
| Nulliparous | 45,398 (42.7) | 163,902 (42.4) |  | 132,901 (41.8) | 76,204 (40.3) |  |
| Multiparous | 60,885 (57.3) | 222,925 (57.6) |  | 185,120 (58.2) | 112,813 (59.7) |  |
| Maternal age at childbirth |  |  | <0.001 |  |  | <0.001 |
| 13-19 | 4498 (4.2) | 23,659 (6.1) |  | 6371 (2.0) | 8238 (4.4) |  |
| 20-26 | 41,604 (39.1) | 177,169 (45.8) |  | 106,933 (33.6) | 86.205 (45.6) |  |
| 27-33 | 46,217 (43.5) | 150,279 (38.8) |  | 149,136 (46.9) | 72.949 (38.6) |  |
| 34- | 13,964 (13.1) | 35,720 (9.2) |  | 55,581 (17.5) | 21,625 (11.4) |  |
|  |  |  |  |  |  |  |
| **Index women** |  |  |  |  |  |  |
| Level of education |  |  | <0.001 |  |  | <0.001 |
| Elementary | 5,577 (6.8) | 18,046 (4.7) |  | 14,622 (4.9) | 15,421 (8.2) |  |
| High school | 28,767 (34.9) | 136,895 (35.8) |  | 114,088 (38.1) | 90,599 (48.2) |  |
| College/University | 48,185 (58.4) | 227,704 (59.5) |  | 170,643 (57.0) | 81,877 (43.6) |  |
| Ever married or cohabiting |  |  | <0.001 |  |  | <0.001 |
| No | 85,539 (80.5) | 144,237 (37.3) |  | 301,034 (94.7) | 122,047 (64.6) |  |
| Yes | 20,744 (19.5) | 242,590 (62.7) |  | 16,987 (5.3) | 66,970 (35.4) |  |
| Birthweight |  |  |  |  |  |  |
| Low birth weight | 4,543(4.3) | 12,466 (3.2) | <0.001 | 11,267 (3.5) | 6,359 (3.4) | <0.001 |
| Very low birth weight^1^ | 511 | 1004 | <0.001 | 1563 | 687 |  |
| Normal birth weight | 101,740 (95.7) | 374,361 (96.8) |  | 306,754 (96.5) | 182,658 (96.6) |  |
| Gestational length |  |  |  |  |  |  |
| Preterm | 9848 (9.3) | 28,656 (7.7) | <0.001 | 30,824 (9.7) | 18,521 (9.8) | 0.218 |
| Very preterm^2^ | 584 | 1150 | <0.001 | 1744 | 745 | <0.001 |
| Term | 96,435 (90.7) | 357,171 (92.3) |  | 287,197 (90.3) | 170,496 (90.2) |  |
| Size for gestational age |  |  |  |  |  |  |
| SGA, 2SD |  |  | <0.001 |  |  | 0.277 |
| No | 101,482 (95.5) | 371,593 (96.1) |  | 410,784 (96.8) | 555,330 (96.4) |  |
| Yes | 4,801 (4.5) | 15,239 (3.9) |  | 13,520 (3.2) | 20,514 (3.6) |  |
| SGA, 10^th^ percentile |  |  | <0.001 |  |  | <0.001 |
| No | 94,777 (89.2) | 346,929 (89.7) |  | 293,824 (92.4) | 173,870 (92.0) |  |
| Yes | 11,506 (10.8) | 39,898 (10.3) |  | 24,197 (7.6) | 15.147 (8.0) |  |
| **Infertility treatment** |  |  |  |  |  |  |
| Q-IVF |  |  | <0.001 |  |  | <0.001 |
| Yes | 3,130 (2.9) | 16,994 (4.4) |  | 1,187 (0.4) | 5,147 (2.7) |  |
| No | 103,153 (97.1) | 369,833 (95.6) |  | 316,834 (99.6) | 183,870 (97.3) |  |
| Donated oocyte^3^ |  |  | <0.001 |  |  | <0.001 |
| Yes | 153 (4.9) | 374 (2.2) |  | 37 (3.1) | 62 (1.2) |  |
| No | 2,977 (95.1) | 16,620 (97.8) |  | 1,150 (96.6) | 5,085 (98.8) |  |

Note: No child = number and percentage of women not registered as mothers in the Multi-Generation Register; Child = number and percentage of women registered as mothers in the Multi-Generation Register; Low birth weight = birth weight <2,500 g; Very low birth weight = birth weight <1,500 g; Preterm = birth before 37th gestational week; Very preterm = = birth before 32th gestational week; Term = birth at 37th gestational week or later, including births after 42nd gestational week; SGA 2 SD= small for gestational age (birth weight <–2 SD of mean weight for gestational age); SGA 10^th^ percentile = small for gestational age (birthweight below the 10^th^ percentile of mean weight for gestational age); Q-IVF = number and percentage of women registered in Q-IVF, the Swedish National Quality Register of Assisted Reproduction.

P-values < .05 are considered to indicate statistical significance.

^1^Subgroup to but also included in Low Birth weight

^2^Subgroup to but also included in Preterm

^3^Limited to women who had undergone infertility treatment.

Table S2. Very preterm birth and very low birth weight in relation to sociodemographic and medical background factors for women born between 1973 and 1983 and between 1984 and 1993 in Sweden.

|  | **Women born 1973-1983** | | | | | | **Women born 1984-1993** | | | | | |
| --- | --- | --- | --- | --- | --- | --- | --- | --- | --- | --- | --- | --- |
|  | **VPT** | | | **VLBW** | | | **VPT** | | | **VLBW** | | |
|  | **No**  **(n=491,376)** | **Yes**  **(n=1,734)** |  | **No**  **(n=491,595)** | **Yes**  **(n=1,515)** |  | **No**  **(n=504,549)** | **Yes**  **(n=2,488)** |  | **No**  **(n=504,518)** | **Yes**  **(n=2,250)** |  |
|  | **n (%)** | **n (%)** | **p-**  **value** | **n (%)** | **n (%)** | **p-**  **value** | **n (%)** | **n (%)** | **p-**  **value** | **n (%)** | **n (%)** | **p-value** |
| **Parents** |  |  | <0.001 |  |  |  |  |  |  |  |  |  |
| Maternal educational level |  |  |  |  |  | <0.001 |  |  | <0.001 |  |  | <0.001 |
| Elementary | 85,514 (19.1) | 358 (24.2) |  | 85.570 (19.1) | 302 (23.2) |  | 54,481 (11.2) | 332 (14.3) |  | 54,535 (11.2) | 278 (13.2) |  |
| High school | 217,703 (48.7) | 713 (48.1 |  | 217,786 (48.7) | 630 (48.5) |  | 247,133 (50.8) | 1251 (53.7) |  | 247,254 (50.8) | 1130 (53.8) |  |
| College/University | 143,752 (32.2) | 410 (27.7) |  | 143,795 (32.2) | 367 (28.3) |  | 184.695 (38.0) | 745 (32.0) |  | 184,747 (38.0) | 693 (33.0) |  |
| Paternal educational level |  |  | 0.013 |  |  | 0.118 |  |  | <0.001 |  |  | 0.147 |
| Elementary | 111,420 (27.8) | 408 (31.1) |  | 111,474 (27.8) | 354 (30.2) |  | 88,245 (19.1) | 494 (22.3) |  | 88,328 (19.1) | 411 (20.4) |  |
| High school | 178,427 (44.5) | 574 (43.8) |  | 178,482 (44.5) | 519 (44.2) |  | 237,262 (51.3) | 1136 (51.2) |  | 237,357 (51.3) | 1041 (51.7) |  |
| College/University | 111,318 (27.7) | 329 (25.1) |  | 111,346 (27.7) | 301 (25.6) |  | 136,710 (29.6) | 589 (26.5) |  | 136,738 (29.6) | 561 (27.9) |  |
| Mother born in a non-Nordic country | 27,293 (5.6) | 123 (7.1) | 0.005 | 27,329 (5.6) | 87 (5.7) | 0.756 | 24,067 /4.8) | 144 (5.8) | 0.018 | 24,085 (4.8) | 126 (5.6) | 0.066 |
| Father born in a non-Nordic country | 23,681 (4.8) | 105 (6.2) | 0.012 | 23,703 (4.8) | 83 (5.5) | 0.211 | 22,714 (4.5) | 142 (5.7) | 0.004 | 22,729 (4.5) | 127 (5.7) | 0.008 |
| Marital status |  |  | <0.001 |  |  | 0.819 |  |  | <0.001 |  |  | <0.001 |
| Married | 299,013 (68.6) | 947 (63.2) |  | 299,098 (68.5) | 862 (67.8) |  | 445,883 (94.5) | 1951 (91.9) |  | 446,039 (94.5) | 1795 (92.5) |  |
| Single | 129,087 (29.6) | 522 (34.8) |  | 129,224 (29.6) | 385 (30.3) |  | 9292 (2.0) | 60 (2.8) |  | 9310 (2.0) | 42 (2.2) |  |
| Divorced/Widow | 8034 (1.8) | 29 (1.9) |  | 8038 (1.8) | 25 (2.0) |  | 16,468 (3.5) | 111 (5.2) |  | 16,476 (3.5) | 103 (5.3) |  |
| Primiparous | 208,470 (42.4) | 830 (47.9) | <0.001 | 208,541 (42.4) | 759 (50.1) | <0.001 | 207,860 (41.2) | 1245 (50.0) | <0.001 | 207,892 (41.2) | 1213 (53.9) | <0.001 |
| Maternal age at childbirth |  |  | <0.001 |  |  | <0.001 |  |  | <0.001 |  |  | <0.001 |
| 13-19 | 28,011 (5.7) | 146 (8.4) |  | 28,050 (5.7) | 107 (7.1) |  | 14,502 (2.9) | 107 (4.3) |  | 14,257 (2.9) | 82 (3.6) |  |
| 20-26 | 218,076 (44.4) | 697 (40.2) |  | 218,155 (44.4) | 618 (40.8) |  | 192,281 (38.1) | 857 (34.4) |  | 192,325 (38.1) | 813 (36.1) |  |
| 27-33 | 195,865 (39.9) | 631 (36.4) |  | 195,944 (39.9) | 552 (36.4) |  | 221,110 (43.8) | 974 (39.1) |  | 221,216 (43.8) | 869 (38.6) |  |
| >33 | 49.424 (10.1) | 260 (15.0) |  | 49,446 (10.1) | 238 (15.7) |  | 76,656 (15.2) | 550 (22.1) |  | 76,720 (15.2) | 486 (21.6) |  |
| **Index women** |  |  |  |  |  |  |  |  |  |  |  |  |
| Level of education |  |  | <0.001 |  |  | <0.001 |  |  | <0.001 |  |  | <0.001 |
| Elementary | 23,502 (5.1) | 121 (7.6) |  | 23,515 (5.1) | 108 (7.8) |  | 29,822 (6.1) | 221 (9.5) |  | 29.832 (6.1) | 211 (10.0) |  |
| High school | 165,005 (35.6) | 657 (41.3) |  | 165,106 (35.6) | 556 (40.1) |  | 203, 628 (42.0) | 1059 (45.5) |  | 203,763 (42.0) | 924 (43.9) |  |
| College/University | 275,078 (59.3) | 811 (51.0) |  | 275,165 (59.3) | 724 (52.2) |  | 251, 471 (51.9) | 1046 (45.0) |  | 251,548 (51.9) | 972 (46.1) |  |
| **Ever married or cohabiting, yes** | 262,564 (53.4) | 770 (44.4) | <0.001 | 262,640 (53.4) | 694 (45.8) | <0.001 | 83,622 (16.6) | 335 (13.5) | <0.001 | 83,654 (16.6) | 304 (13.5) | <0.001 |
| **Child, yes** | 385,677 (78.5) | 1150 (66.3) | <0.001 | 385,823 (78.5) | 1004 (66.3) | <0.001 | 188.272 (37.3) | 745 (29.9) | <0.001 | 188,330 (37.3) | 687 (30.5) | <0.001 |
| **Infertility treatment** |  |  |  |  |  |  |  |  |  |  |  |  |
| Q-IVF, yes | 20,074 (4.1) | 50 (2.9) | 0.012 | 20,080 (4.1) | 44 (2.9) | 0.020 | 6322 (1.3) | 12 (0.5) | <0.001 | 6321 (1.3) | 13 (0.6) | 0.004 |
| Donated oocyte^1^, yes | 5252 (2.6) | 2 (4.0) | 0.378 | 523 (2.6) | 4 (9.1) | 0.007 | 99 (1.6) | 0 (0.0) | 1.000 | 99 (1.6) | 0 (0.0) | 1.000 |

Note: VPT = very preterm (birth before 32th gestational week); VLBW = low birth weight (<1,500 g); Marital status: Married = ever registered as married in the Total Population Register (TPR); Single = never registered as married or cohabiting in the TPR; Q-IVF = number and percentage of women registered in Q-IVF, the Swedish National Quality Register of Assisted Reproduction. P-values < .05 are considered to indicate statistical significance. ^1^Limited to women who had undergone infertility treatment.

Figure S1. Directed acyclic graph on potential confounding and mediation.


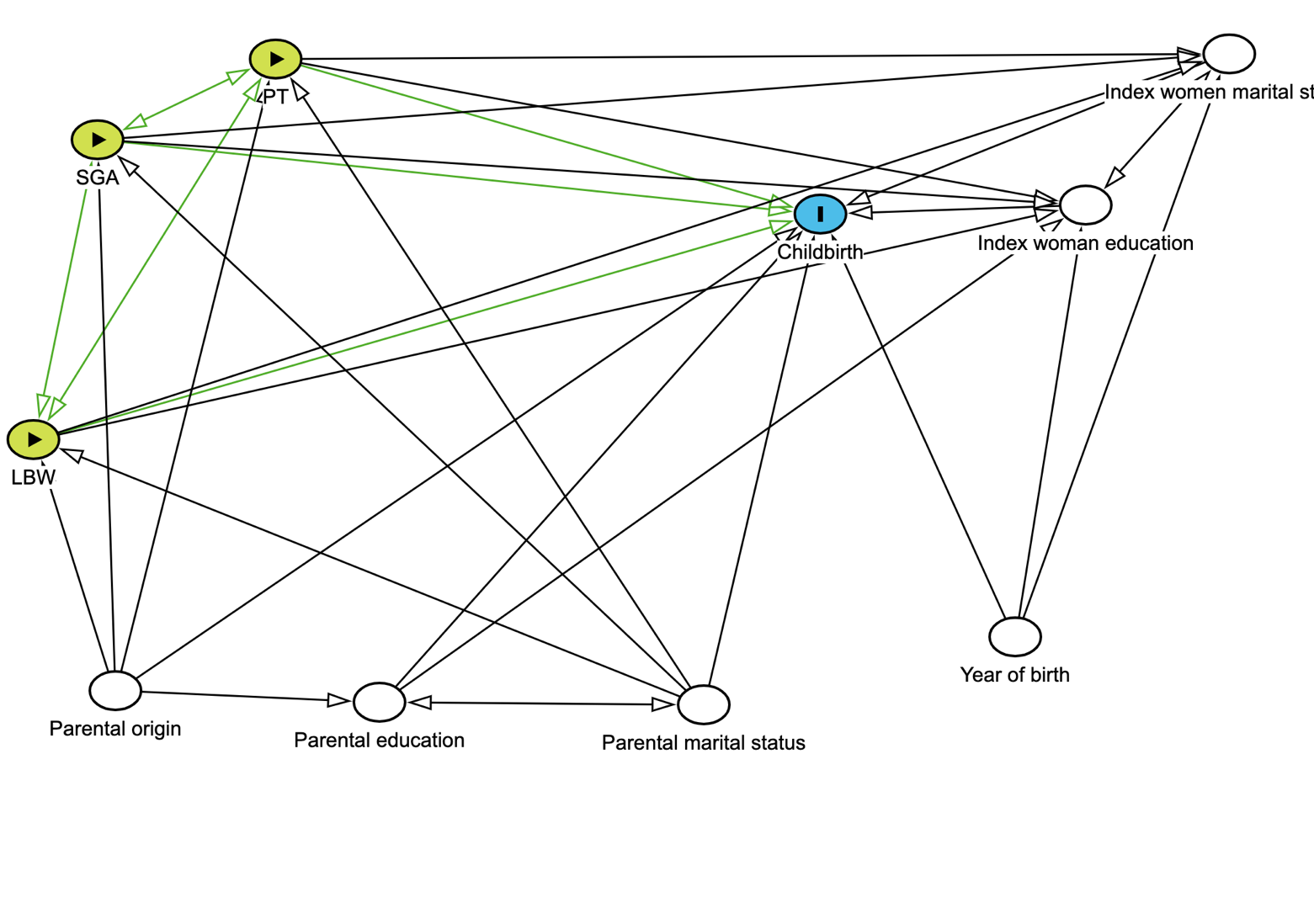

Supplement: Supplementary file 1 — Table S1: Descriptive data of women born between 1973 and 1983 and between 1984 and 1993 in Sweden. Table S2: Very preterm birth and very low birth weight in relation to sociodemographic and medical background factors for women born between 1973 and 1983 and between 1984 and 1993 in Sweden. Figure S1: Directed acyclic graph on potential confounding and mediation. [file BJO-133-165-s001.docx]
